# Navigating Host Immunity and Concurrent Ozone Stress: Strain‐Resolved Metagenomics Reveals Maintenance of Intraspecific Diversity and Genetic Variation in *Xanthomonas* on Pepper

**DOI:** 10.1111/eva.70069

**Published:** 2025-01-14

**Authors:** Amanpreet Kaur, Ivory Russell, Ranlin Liu, Auston Holland, Rishi Bhandari, Neha Potnis

**Affiliations:** ^1^ Department of Entomology and Plant Pathology Auburn University Auburn Alabama USA

**Keywords:** genetic variation, global environmental change, pathogen evolution, plant disease, resistance durability

## Abstract

The evolving threat of new pathogen variants in the face of global environmental changes poses a risk to a sustainable crop production. Predicting and responding to how climate change affects plant‐pathosystems is challenging, as environment affects host–pathogen interactions from molecular to the community level, and with eco‐evolutionary feedbacks at play. To address this knowledge gap, we studied short‐term within‐host eco‐evolutionary changes in the pathogen, *Xanthomonas perforans* , on resistant and susceptible pepper in the open‐top chambers (OTCs) under elevated Ozone (O_3_) conditions in a single growing season. We observed increased disease severity with greater variance on the resistant cultivar under elevated O_3_, yet no apparent change on the susceptible cultivar. Despite the dominance of a single pathogen genotype on the susceptible cultivar, the resistant cultivar supported a heterogeneous pathogen population. Altered O_3_ levels led to a strain turnover, with a relatively greater gene flux on the resistant cultivar. Both standing genetic variation and de novo parallel mutations contributed toward evolutionary modifications during adaptation onto the resistant cultivar. The presence of elevated O_3_, however, led to a relatively higher genetic polymorphism, with random and transient mutations. Population heterogeneity along with genetic variation, and the promotion of interdependency are mechanisms by which pathogen responds to stressors. While parallel mutations may provide clues to predicting long‐term pathogen evolution and adaptive potential. And, a high proportion of transient mutations suggest less predictable pathogen evolution under climatic alterations. This knowledge is relevant as we study the risk of pathogen emergence and the mechanisms and constraints underlying long‐term pathogen adaptation under climatic shifts.

## 1 Introduction

Plants and pathogens constantly engage in a co‐evolutionary arms race, where interaction of the plant resistance (R) gene with the corresponding pathogen avirulence (Avr) gene triggers the dynamics of avoidance of detection and balancing fitness penalties, facilitating long‐term maintenance of polymorphism in the Avr and R genes (Bakker et al. 2006; Karasov et al. 2014; Leach et al. 2001; Leonard and Czochor 1980; Mauricio et al. 2003; Van der Plank 1968; Vera Cruz et al. 2000). Plant resistance is a sustainable and cost‐effective approach to managing pathogen outbreaks effectively in modern agricultural systems; however, the durability of the resistance depends on the nature of the resistance genes. Qualitative resistance breaks down rapidly within 3–5 years. Quantitative resistance, on the other hand, is deemed durable due to the slower adaptation of pathogen to the polygenic traits encompassing additive multiple small‐effect quantitative trait loci (QTLs) (Brown and Rant 2013; Cheng et al. 2013; Janda et al. 2019; McDonald and Linde 2002; Poland et al. 2009; St. Clair 2010). However, this process of pathogen adaptation to quantitative resistance is poorly understood (Caffier et al. 2016; Corwin and Kliebenstein 2017; McDonald and Linde 2002; Pariaud et al. 2009; St. Clair 2010).

Climatic fluctuations further add complexity to these eco‐evolutionary dynamics of host–pathogen interactions and add uncertainty to the outcomes of resistance management. Climatic alterations can influence the stability of gene‐for‐gene interactions and can change the direction of selection (Cable et al. 2017) with unexpected outcomes (Mostowy and Engelstädter 2011). Global environmental changes can alter the evolution of virulence (Kutzer and Armitage 2016; Schmid‐Hempel 2003), reinforce the selection on immunity genes, and accelerate pathogen adaptation, thereby increasing plant disease risk (Laine 2023). Climate sensitivity of basal resistance (Janda et al. 2019), as well as both qualitative and quantitative resistance (Cheng et al. 2013; Cohen et al. 2017; Onaga et al. 2017; Webb et al. 2010), has been noted, with few noted as climate resilient (Cohen et al. 2017). Climate sensitivity of plant pathogens within hosts is unclear (Huot et al. 2017; Onaga et al. 2017; Velásquez, Castroverde, and He 2018), and unlike animal pathogens (Shapiro and Cowen 2012), little is known about virulence modulation under abiotic stress.

Among the abiotic stressors brought about by global changes, ozone pollution has been a growing concern (Lefohn et al. 2018; Vingarzan 2004). Over the past three decades, increase in tropospheric ozone (O_3_) levels has been noted to be 2%–12%, with peak concentrations in summer growing seasons (Ainsworth 2017; Brauer et al. 2016; Tan and Wang 2022). Ozone concentrations above 40 parts per billion (ppb) are phytotoxic (Ainsworth 2017, Saxena et al. 2020). It is predicted that daily 8‐h maximum O_3_ levels will increase from 31–79 ppb (from 2012) to 30–87 ppb in 2050 (Pfister et al. 2014). Ozone stress has been demonstrated to impair photosynthetic efficiency due to cellular damage brought about by reactive oxygen species (ROS), a breakdown product of O_3_, once taken up by stomata. O_3_ can also induce stomatal sluggishness and ultimately impact carbon assimilation, alter plant physiology, and overall productivity (Paoletti, Grulke, and Matyssek 2019). Additionally, it can change the host immunological response, causing signaling cascades and other cellular reactions that damage plant cuticles oxidatively and make plants more susceptible to disease (Burkart et al. 2013; Chen, Frank, and Long 2009; Grantz and Vu 2012; Pellegrini et al. 2013; Romero et al. 2020; Vahala et al. 2003; Violini 1995). In contrast, negative impact of O_3_ on disease development has also been observed in case of strawberry pathogen (Laurence and Wood 1978). Thus, pathogen lifestyle may influence the overall disease outcome when plants are challenged with abiotic stressor. Further work is needed to evaluate how O_3_ can impact pathogen and the host individually, as well as trade‐offs faced by the plant when responding to the simultaneous stressors. Field pathogenomics on spatiotemporal samples combined with population genetics has been the approach to studying pathogen adaptation, although the major focus has been the host selection pressure (Badouin et al. 2017; Grünwald, McDonald, and Milgroom 2016; Hartmann and Croll 2017; Thilliez et al. 2019). These studies have informed that various mechanisms of genetic variation are in place, such as allelic polymorphisms in pathogen avirulence genes (Gassmann et al. 2000; Gautier et al. 2023; Huang et al. 2014, 2019; Martynov et al. 2019; Yang et al. 2019); genome rearrangements, with identification of rapidly evolving genomic regions carrying clusters of virulence determinants (Frantzeskakis et al. 2020; Rouxel et al. 2011; van Dam et al. 2016; Whisson et al. 2012); gene gain/loss bringing in genetic novelty (Hartmann and Croll 2017; Tsushima et al. 2019; Yoshida et al. 2016); or sexual recombination facilitating rapid fixation of beneficial mutations (Grandaubert, Dutheil, and Stukenbrock 2019). Distinct genomic compartments were found to have different rates and modes of evolution (Möller and Stukenbrock 2017; Montoya and Raffaelli 2010). Genomic signatures in the pathogen indicate the influence of both directional and balancing selections in adaptation (Brunner and McDonald 2018; de Vries, Stukenbrock, and Rose 2020; Hall et al. 2020; Poppe et al. 2015). Although the impact of host selection pressure in driving genomic changes has been explored, the impact of global environmental changes driving pathogen evolution is limited (Nnadi and Carter 2021; Wu et al. 2020).

**FIGURE 1:**
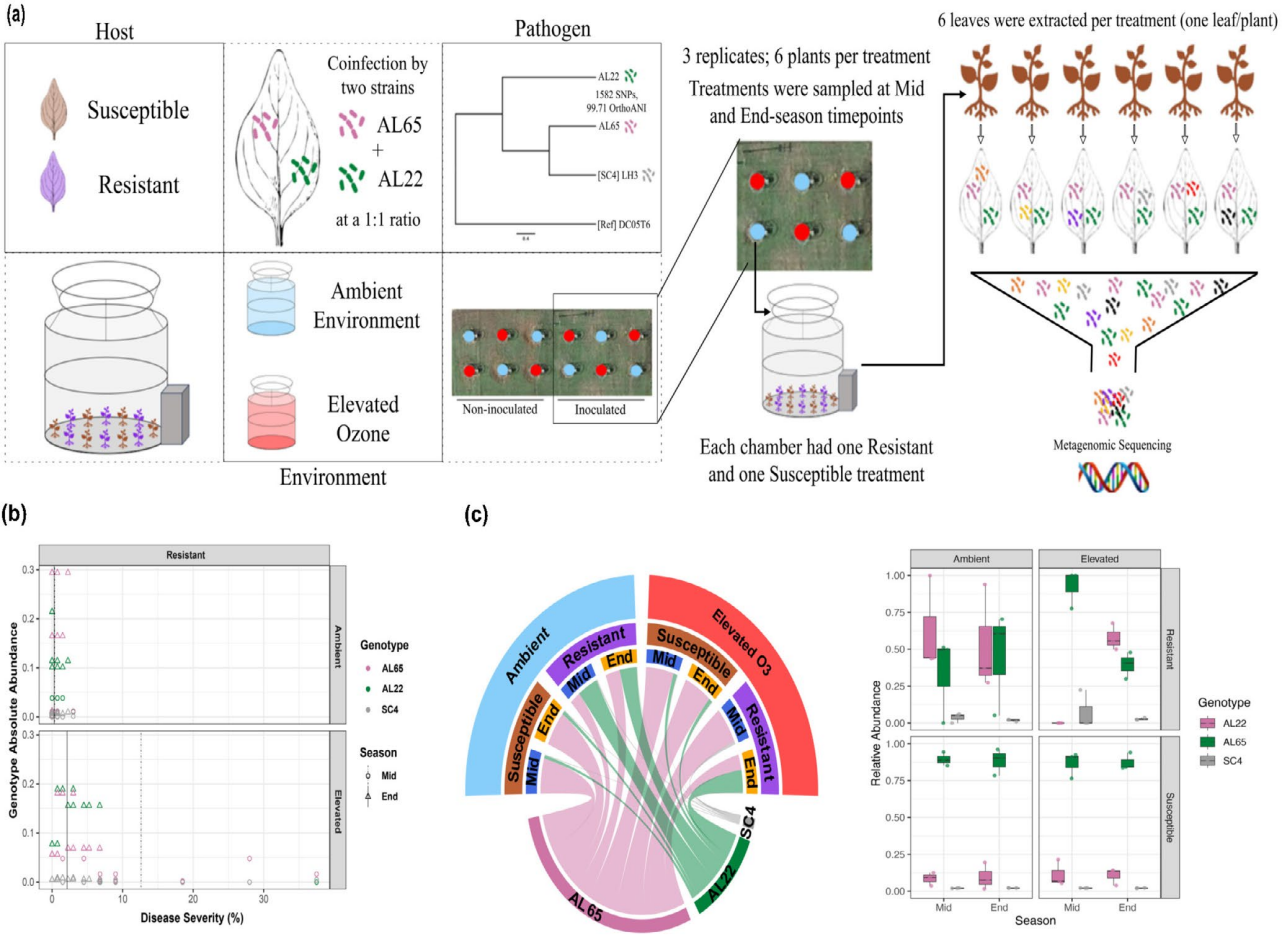
Experimental setup and pathogen population and disease dynamics. (a) We setup an experiment in which we inoculated two *Xanthomonas perforans* (*Xp*) *strains* at a 1:1 ratio onto susceptible and resistant plants and exposed them to ambient or elevated levels of O_3_ within open‐top chambers. Out of the 12 open‐top chambers, 6 were non‐inoculated controls, and 6 were inoculated (highlighted inside the black square box); additionally, six were exposed to elevated O_3_ (presented with red colored circles) while the remaining six were not exposed (shown with sky‐blue colored circles). Each chamber contained 12 plants, making up 2 treatments, half susceptible cultivars and half resistant, with each cultivar set being a treatment, respectively. At mid‐ and end‐season time points, three samples per treatment were collected, with each sample comprising leaves picked randomly from six plants per chamber, and their metagenomic DNA was extracted and sequenced. The relative abundance of inoculated pathogen genotypes AL22 and AL65 was traced. We also observed evidence of natural infection by a representative of sequence cluster 4 (SC4), although it did not show an increase in abundance over time. (b) Absolute abundance of *Xp* genotypes as compared to disease severity during the mid‐ and end‐season sampling time points for the resistant cultivar under ambient versus elevated O_3_ levels. The mid‐ and end‐season disease severity averages vary from ambient to elevated O_3_. Absolute abundance by disease severity for susceptible cultivar is presented in Figure S2. (c) Relative abundance of *Xp* genotypes as averages in the leftmost circular chord diagram and raw values in the rightmost bar plot with individual points showing the values from each sample.

We set out to understand the impact of elevated O_3_ on plant‐pathogen interactions using an experimental setup in OTCs. We inoculated resistant and susceptible pepper cultivars with *Xanthomonas perforans* (*Xp*, also referred to as *X. euvesicatoria* pv. *perforans*) and exposed them to ambient and elevated ozone (O_3_) conditions in OTCs (Figure 1a, Figure S1) and recorded disease severity levels twice during the season. Resistant cultivar used in this experiment contains *bs5* resistance gene, which imparts non‐hypersensitive type of resistance and is considered durable against currently isolated bacterial spot pathogens (Stall, Jones, and Minsavage 2009; Subedi et al. 2024; Szabó et al. 2023). This resistance gene is now cloned, and the mechanism of resistance has been proposed to be involvement of PAMP‐triggered immunity and ROS generation (Subedi et al. 2024). The experimental design used here allowed us to evaluate climate sensitivity of this currently deployed resistance in commercial pepper variety when inoculated with *Xanthomonas*. The elevated O_3_ level did not influence disease severity levels on the susceptible cultivar. However, under elevated O_3_ levels, the resistant cultivar showed significantly higher disease severity scores, with an average increase of 12% during mid‐season and 2% during end‐season compared to an ambient environment (Figure 1b; Bhandari et al. 2023). This increase in disease severity under elevated O_3_ could be due to direct or indirect effects of climate on pathogen infectivity or altered ecological interactions among pathogen genotypes, or a combination of all these factors. Other explanations for this increased disease severity could be alteration of plant response toward pathogen or altered microbiome‐mediated protection leading to increased susceptibility under elevated O_3_. We observed alteration of the physiological response of the resistant cultivar with changes in stomatal responsiveness under elevated O_3_ and simultaneous challenge with pathogen and this led to reduced stomatal conductance and carbon assimilation rate on resistant cultivar (Modelski et al. 2024). Our observations of distinct responses of microbial communities to individual and simultaneous stressors and altered microbiome stability may suggest that microbiome‐mediated protection might be compromised under elevated O_3_ (Bhandari et al. 2023).

Here, we focused on investigating the influence of altered O_3_ levels on pathogen from the gene to population level and whether ecological fluctuations and/or evolutionary modifications in the pathogen population could explain the increase in disease severity observed on the resistant cultivar under elevated O_3_ conditions. Most data on pathogen evolution come from temporal field surveys coupled with isolate genome sequencing, which may carry inherent culturing bias (Newberry et al. 2020) and often do not dissect the impact of complex host, pathogen, and climate interactions. The strain‐resolved metagenomics approach overcomes the bias of selecting only a dominant pathogen lineage and provides a comprehensive view of pathogen variants, including low‐abundant variants, that may hold the potential to cause outbreaks in a suitable environment (Newberry et al. 2020). Here, we used strain‐resolved metagenomics to investigate within‐host dynamics, with a high resolution into genetic processes shaping rapid pathogen evolution during a single growing season. We hypothesized that pathogen adaptation to the simultaneous challenge of host defense and elevated O_3_ would involve population heterogeneity and a relatively high rate of evolution. Our study system allowed us to test this hypothesis, since we had inoculated the plants with two closely related genotypes of *Xp* to mimic the co‐occurrence observed in the recent field outbreaks (Newberry et al. 2023). The two selected genotypes belong to two different sequence clusters (SCs), namely, SC6 (AL65, isolated from a susceptible pepper cultivar) and SC2 (AL22, isolated from a resistant pepper cultivar). The population genetic methods applied to the high‐resolution shotgun metagenome data allowed us to capture the population dynamics at the intra‐subspecific level of phylogenetic resolution, learn about the generation and maintenance of genetic variation and assess whether the variation could explain the increased susceptibility on resistant cultivar under elevated O_3_. Overall, our data provide an understanding of how population dynamics both shape and are shaped by evolutionary processes and how pathogen population may respond to future climatic conditions.

## 2 Methods

### 2.1 Experimental Setup and Data

An experimental setup in the OTC at the field site is described in Figure 1a. In this study, both susceptible (Early Cal Wonder) and resistant (PS 09979325, contains recessive resistance *bs5* gene) cultivars of pepper were used (Modelski et al. 2024; Monroy 2019). Briefly, 4‐ to 6‐week‐old resistant and susceptible pepper plants were inoculated with a mixture (1:1) of two *Xp* strains, AL65 (SC6) and AL22 (SC2), to mimic the pathogen heterogeneity observed in the field conditions (Newberry et al. 2020) and monitored for disease development throughout the growing season of 2021. Plants were fumigated for 8 h per day between the hours of 10 a.m. and 6 p.m. The average ambient O_3_ concentration was 30.6 ppb, and the averaged elevated O_3_ concentration was 90.3 ppb (Bhandari et al. 2023). These levels are comparable to those that are predicted to occur under future climatic conditions (Pfister et al. 2014). We randomly collected one leaf per plant within each OTC and combined the leaves from six plants of each cultivar of each chamber. Since the pathogen was the main focus of this investigation, we only took into account samples from six chambers that had been inoculated with the pathogen (Bhandari et al. 2023). The phyllosphere samples were obtained from each treatment in triplicates during the mid‐season (June 18) and end‐season (July 29) timepoints. The leaf washings from these samples obtained upon sonication were subjected to total DNA extraction, followed by metagenome sequencing (Bhandari et al. 2023). Further, BBDuk (Bushnell 2020) to trim the raw reads for quality, and KneadData (The Huttenhower Lab 2020) was used to remove host contamination utilizing the genome of pepper cv. 59 (GCA_021292125.1) as a reference.

### 2.2 Abundance of Inoculated Strains

We assessed the influence of host genotype and elevated O_3_ on strain dynamics by tracing the relative and absolute abundance of two inoculated pathogen genotypes using StrainEst (Albanese and Donati 2017). Briefly, the reference single‐nucleotide variants (SNVs) profiles representative of six lineages, referred as SCs within *Xp* (Newberry et al. 2020) were constructed. Representative strains chosen for each SC were GEV993 (SC1), AL22 (SC2), AL57 (SC3), LH3 (SC4), AL33 (SC5), and AL65 (SC6). An ordered vector of SNVs was obtained from the SNV profile, and the SNV matrix of the species was constructed. For example, the SNP distance between two co‐inoculated strains, SC2 and SC6 is 1583; that between SC6 and SC4 is 425, and that between SC2 and SC4 is 9227. This SNV matrix allowed profiling of each SC in the metagenomic samples, allowing evaluation of natural infection by SC4, rather than limiting strain profiling of the inoculated strains, belonging to SC2 and SC6. The metagenomic reads were aligned to extract the frequency of occurrences of each of the possible alleles from the aligned reads, low coverage sites were removed, and the frequency profile for each metagenome was obtained using the LASSO regression model (Albanese and Donati 2017). This provided a relative abundance of different SCs in each sample. We calculated an estimate of the absolute abundance of each SCs by multiplying its relative abundance by ng of DNA per mg of sample.

### 2.3 Nonredundant Pangenome of *Xp* Inoculated Strains

A nonredundant pangenome of *Xp* strains AL65 and AL22 was built using SuperPang (v0.9.4beta1) (Puente‐Sánchez et al. 2022). This pangenome was then used as a reference for the rest of the analyses. A total of 86.5% of this nonredundant pangenome is the core genome, whereas 6% is unique to AL65, and 7.5% of the sequence is unique to AL22.

### 2.4 Identification and Removal of Blacklisted Genes From Metagenomic Reads

Since we were interested in inferring evolutionary changes in the pathogen, we implemented filters to avoid read stealing and donating. The read stealing and/donating may occur from the erroneous matches to the orthologous genes present in the closely related co‐occurring species in our metagenome samples. Such genes that are shared with high homology across species of interest, that is, *X. perforans* in this study, and the other resident microbiome community members are referred to as blacklisted genes. To identify such blacklisted genes, we first identified phyllosphere community members present in our samples using “run_midas.py species” command that is a part of MIDAS (v1.3.2). This step utilizes MIDAS (v1.3.2) default database as a reference (consisting of 31,007 bacterial reference genomes organized into 5952 species groups) (Nayfach 2015/2022). The species with mean abundance > 0 in our metagenome samples were identified (Table S1). Next, the gene sequences from these co‐occurring species were compiled. We then used the nonredundant *Xp* pangenome to format the MIDAS‐compatible custom database for species of our interest (*Xp*) and subjected it to BLAST (blast+) with the concatenated gene sequences from the species with a mean abundance > 0 (listed in Table S1) from the previous step. The gene sequences from the phyllosphere species database with a similarity of 97% with *Xp* pangenome were identified as blacklisted genes (Table S2).

### 2.5 Evolutionary Modifications

The MIDAS package was used to find alleles with their frequencies for different SNV sites throughout the pangenome. First, “run_midas.py snps” script was run on quality filtered raw reads of individual samples. Then, to find major and minor alleles in the population, all samples/replicates of each treatment/condition were merged using “merge_midas.py snps” script with the default settings. Building upon this, the methodology involved further steps tailored to find the patterns of standing genetic variation and de novo mutations (associated with Figure 3). Here, we focused exclusively on biallelic sites, excluding multi‐allelic ones. To calculate the major allele frequency, we subtracted the minor allele frequency from 1. At first, we identified sites where minor alleles present during the mid‐season shifted to become major alleles by the end of the season in different treatments. We then examined allele frequencies further, selecting only those sites where the allele frequency was less than 0.2 during mid‐season, and exceeded 0.8 by the end of the season for the same allele (Figure 3a). Following that, MUMmer (v3.0) (Kurtz et al. 2004) was used to identify genomes associated with specific alleles within the population. Using the nonredundant pangenome of *Xp* as the reference, we mapped the genomes of *Xp* strains AL22, AL65, and LH3 to determine SNP locations with the allele information at the corresponding sites in the reference genome. Then, these identified SNPs were aligned with the major and minor alleles at sites undergoing allele shifts (Table S4). According to Figure 1, we did not find the presence of other *Xp* lineages (other than our inoculated strains, AL22, AL65, and low levels of SC4); thus, we only considered these three genomes. Similarly, to identify de novo mutations, we used MUMmer and focused primarily on the major alleles present in the mid‐ and end‐season populations (Figure 3, Tables S5 and S6). We mapped all three genomes (AL65, AL22, and LH3) and considered an allele as de novo if it did not match any SNP identified from the MUMmer output.

From all the above‐identified sites, we excluded all the SNV sites associated with blacklisted genes (Table S2). Along with this, we examined the read depth of each sample/replicate within a treatment, considering only those sites that are present among at least two replicates and have a minimum read depth of 10. The alleles or SNVs were reported as “parallel,” which were present in at least two replicates. We normalized the site counts by dividing them by the average mean coverage or sequencing depth value per treatment.

### 2.6 Genetic Differentiation and Selection Pressure

The identified blacklisted genes (in Section 2.4, Table S2) were removed from all the metagenome sample reads using bbduk (v37.36) (Bushnell 2014). The number of reads removed due to blacklisted genes is documented in Table S3. The reads obtained after filtering for blacklisted genes were used for downstream analyses below. We mapped the samples to the pangenome using BWA‐MEM (v0.7.12) (H. Li and Durbin 2009), followed by the removal of low‐quality alignments and duplicate reads using Samtools (v1.11) (Li et al. 2009) and Picard (v1.79) (Picard n.d.), respectively. The pairwise fixation index (*F_st_*) was calculated for each 1 kbp sliding window using PoPoolation2 (v1201) with the following parameters: ‐‐min‐count 2, ‐‐min‐coverage 4, and ‐‐max‐coverage 120. To calculate within‐host nucleotide diversity (*π*), we used MetaPop (v1.0) (Gregory et al. 2022). We employed MetaPop's local alignment algorithm, which normalizes diversity estimates by dividing them by the genome length to account for uneven coverage across all samples. Additionally, it excludes SNV positions not covered in the genome length and sets a PHRED score threshold of > 20 for local SNV calls. For statistical analysis, the Shapiro test was run for the distribution of our dataset; the Kruskal–Wallis rank sum test and Dunn test were performed for overall and pairwise comparisons, respectively. A *p*‐value of < 0.05 was considered statistically significant.

To measure the selective pressure on the genome of pathogen population by calculating Tajima's *D* on 1 kbp window. We first piled up the reads from all the replicates for each treatment using samtools, then used PoPoolation (v1.2.2) to calculate Tajima's *D* values for each 1 kbp window of genome with the following parameters: ‐‐min‐count 2, ‐‐min‐coverage 4, ‐‐window‐size 1000, and ‐‐step‐size 1000. Later, we chose those windows where our selected loci (from Section 3.3) existed in the genome and associated with the genes coded in those regions.

### 2.7 Gene Gain and Loss

We used MIDAS (v1.3.2) to estimate gene changes in the pathogen population (associated with Figure 4). In this analysis, we refer to the gene flow within the microbial population pool, where gene flow may have occurred toward the *Xp* population during the growing season. We used "run_midas.py genes" script on each sample, then merged all samples within each treatment using "merge_midas.py genes" script with default settings. Whenever a gene has a minimum copy number of 0.35, it is considered present or otherwise absent (Garud and Pollard 2020). Blacklisted genes were removed later from the output. All gene annotations were performed using eggNOG‐mapper v2 (Cantalapiedra et al. 2021). Later, we mapped the genes with the genome of both strains using Blast to find the accessory genes among both strains.

## 3 Results

### 3.1 Pathogen Population Dynamics Was Host‐Genotype‐Dependent, and Elevated O_3_ Altered Strain Dynamics on the Resistant Cultivar

To understand the response of the pathogen population during adaptation to the resistant host and under altered O_3_ levels, we traced the abundance of the co‐inoculated pathogen genotypes across treatments over time. Although there was an incidence of natural infection by the SC4 genotype, this genotype did not increase in frequency during the growing season. The presence of host resistance had a significant effect on the relative and absolute abundance of the inoculated genotypes AL65 (*p* < 0.01, Kruskal–Wallis) and AL22 (*p <* 0.01, Kruskal–Wallis; Figure 1c, Figures S2 and S3). Strain AL65 outperformed strain AL22 on the susceptible cultivar throughout the growing season, regardless of the O_3_ level. On the other hand, strain‐level heterogeneity was observed on the resistant cultivar, although the host × environment interaction likely influenced the strain dynamics. Strain AL22 (isolated initially from a resistant cultivar) maintained a higher population on the resistant cultivar under an ambient environment during mid‐season. Despite the lower absolute abundance of *Xanthomonas* during the mid‐season on the resistant cultivar, it is worth noting that the two strains showed preferences in colonization under different conditions, with AL65 being dominant under elevated O_3_ and AL22 under ambient conditions. By the end‐season, both strains coexisted on the resistant cultivar regardless of the environmental conditions (Figure 1c, Figures S2 and S3).

### 3.2 Higher Genetic Differentiation in the Pathogen Population Was Observed on the Resistant Cultivar Under Elevated O_3_

**FIGURE 2:**
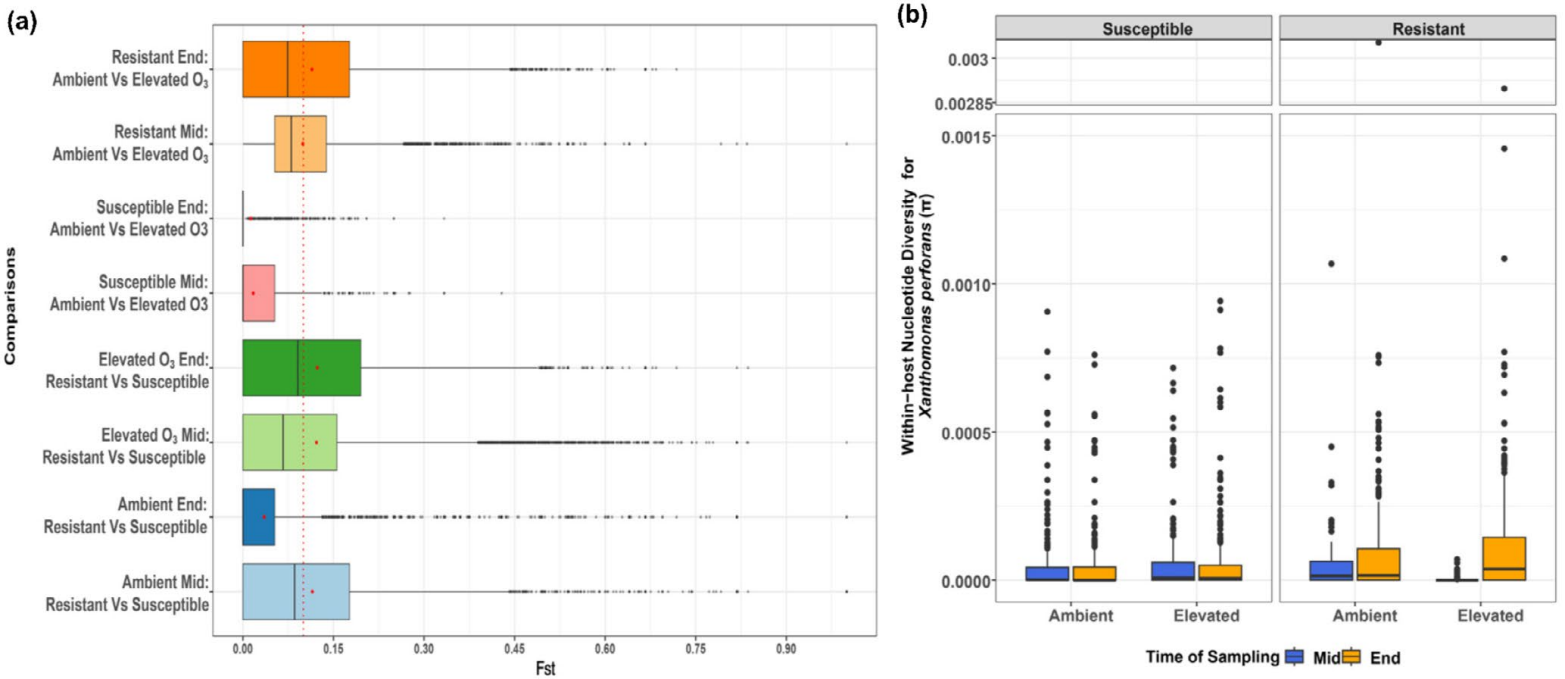
The pathogen population adapted to the resistant cultivar under elevated O_3_ with higher genetic differentiation. (a) Boxplots showing pairwise host comparisons with *F*_ST_ (Fixation Index) values (calculated using PoPoolation2) for 1 kbp window. The red color dashed line indicates *F*_ST_ = 0.15 which is considered as substantial differentiation between compared treatments' populations moderate differentiation. *F*_ST_ values greater than 0.15 are considered high genetic differentiation between comparisons. (b) Boxplot showing within‐host nucleotide diversity (*π*) of the *Xp* population together for different treatments and values for individual replicates are shown in Figure S4.

Next, we investigated whether genetic differentiation in the pathogen population may account for the greater disease severity values seen under elevated O_3_ on the resistant cultivar since the absolute abundance of *Xanthomonas* could not explain this observation (Figure 1b; Bhandari et al. 2023). Here, we hypothesized that adaptation to the stressors (host resistance or elevated O_3_ in either host background) is reflected in higher genetic differentiation in the pathogen population. According to pairwise host comparisons, the population recovered from the resistant cultivar presented greater genetic divergence compared to that from susceptible cultivar under elevated O_3_. Sampling time also influenced genetic differentiation levels, with mid‐season populations having higher *F*_*ST*_‐values than end‐season. However, adaptation to the resistant host under elevated O_3_ resulted in greater population divergence at the end‐season (Figure 2a). These observations for genetic differentiation were further confirmed with within‐host nucleotide diversity values. We observed higher but variable within‐host nucleotide diversity (*π*) values (Figure 2b, Figure S4) in the pathogen population from the resistant cultivar as compared to the susceptible cultivar irrespective of environmental conditions (*p*_*π*_ < 0.001). End‐season pathogen populations from the resistant cultivar under elevated O_3_ conditions had greater within‐host nucleotide diversity values and mutation rates than mid‐season populations (*p*_*π*_ < 1e‐12). Interestingly, the nucleotide diversity for pathogen populations from the resistant cultivar during mid‐season was significantly higher under ambient O_3_ conditions as compared to under elevated O_3_ (*p*_*π*_ < 1e‐5), but there was no significant difference in the within‐host nucleotide diversity values by the end‐season (Figure 2b). Next, we measured the extent of within‐host polymorphism in the pathogen population by identifying alleles displaying intermediate SNV frequency (0.2–0.8) and classifying them as synonymous or non‐synonymous mutations (Figure S5). We compared normalized SNV counts (relative to the absolute abundance of *Xp* population) across treatments. Overall, greater SNV counts were detected on the resistant cultivar compared to the susceptible cultivar under both environments (*p*_(ambient)_: mid < 0.0001, end < 0.01; *p*_(elevated O3)_: mid < 0.05, end < 0.001). There were no significant differences between non‐synonymous and synonymous mutations across different conditions.

### 3.3 The Genetic Differentiation Observed Across Cultivars and O_3_ Conditions Is due to Standing Genetic Variation and De Novo Mutations

**FIGURE 3:**
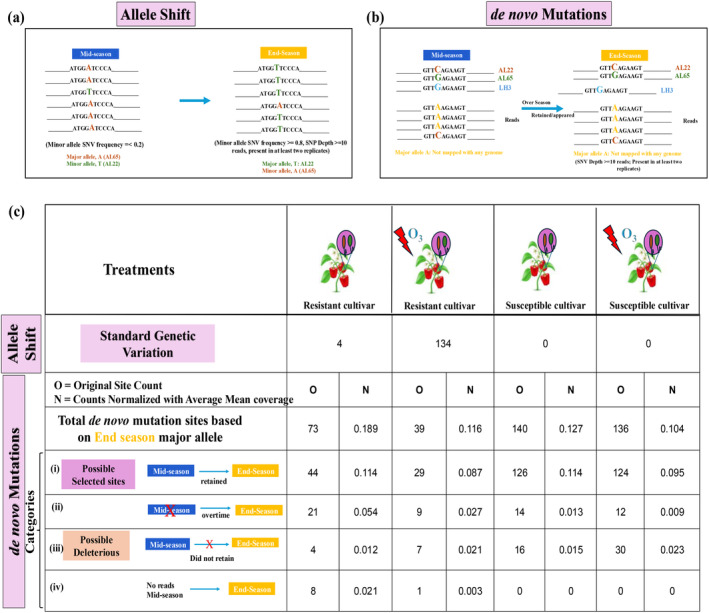
Evolutionary modifications with strain replacement were observed in pathogen population from the resistant cultivar under elevated O_3_. (a) Visual representation of the allele shift (or standard genetic variation), where the minor allele (T) at the highlighted SNV site had a frequency of less than 0.2 during the mid‐season and the allele is mapped to AL22 (also confirmed by mapping with AL65 and SC4). However, by the end‐season, frequency of allele T at the same SNV site was increased to more than 0.8, that is, allele T has replaced A and present in more than 80% of the reads of the pathogen population in the respective sample. (b) Visual representation of de novo mutations, in which the major alleles for mid‐ and end‐season were mapped to the reference genomes of *Xp* (AL22, AL65, and SC4) for all sites, and those that did not match any of the genomes were then pooled out, called them as de novo mutations. (c) Table presenting counts for the allele shift, and total de novo *mutations* parallel (in two or more replicates) for different treatments, which were further separated into different categories: (i) possibly selected sites, which were present in mid‐season and retained in the pathogen population by the end‐season, (ii) those that were appeared in the population over time or by end‐season, (iii) possible deleterious mutations, which were not retained from mid‐season to end‐season in the pathogen population, and (iv) and where no mid‐season information is available because of low sequencing depth. (Note: We normalized the counts by dividing them by the average mean coverage or sequencing depth value per treatment. In all the count estimations, only those sites with SNV depth of a minimum of 10 reads during the end‐season with their presence in at least two replicates for all the treatments (i.e., parallel mutations) were considered. Additionally, sites belonging to blacklisted genes were removed from these calculations).

The greater proportion of within‐host polymorphism observed on the resistant cultivar (Figure 2b) may be attributable to standing genetic variation and/or de novo mutations. Indeed, the results on strain dynamics indicated the coexistence of two pathogen genotypes on resistant cultivar and shifts in their frequencies under elevated O_3_ (Figure 1c), thus contributing to standing genetic variation (Figure 1). However, the contribution of evolutionary modifications that arose de novo during the growing season cannot be ruled out. Our experimental design involving co‐inoculating two closely related pathogen genotypes (1583 SNVs apart) allowed us to assign the alleles to the parent genomes and tease apart SNVs arising during the growing season. To understand the contribution of allelic shifts that result from standing genetic variation, we focused on identifying SNVs with a frequency of ≤ 0.2 during mid‐season but shifted to ≥ 0.8 by the end‐season (Figure 3a). These rare sweeping alleles may have adaptive potential. These alleles matched the AL65 and AL22 parent genomes, thus indicating the contribution of standing genetic variation. As expected from the strain dynamics data, no such alleles were found in the susceptible cultivar, regardless of environmental conditions (Figure 3c). However, a larger proportion of parallel allele shifts leading to the fixation of AL22 alleles (134 alleles) was observed on the resistant cultivar under elevated O_3_ (Table S4). These were associated with genes involved in host immune suppression or enhanced nutrient acquisition or assimilation, such as TonB‐dependent receptor, outer membrane protein, XopV effector protein, and acid phosphatase (Table S4). In contrast, the contribution of the allele shift leading to fixation (four alleles) was much smaller in pathogen on the resistant cultivar under the ambient environment, with two alleles belonging to AL65 in the gene encoding the XopAD type III effector protein (Table S4).

To identify if any SNVs resulted from de novo mutations, we identified sites with major alleles that did not match AL65 or AL22 parents or SC4 (natural infection) (Figure 3b). Among these de novo mutations, we first focused on those observed as novel major alleles in the end‐season samples, which were parallel in at least two replicates with a minimum read depth of 10. Owing to variable sequencing depths across metagenome data from resistant and susceptible cultivars, we normalized the SNV counts with the average mean coverage/sequencing depth value per treatment. The number of normalized parallel de novo mutations was highest in the pathogen population recovered from the resistant cultivar under ambient conditions (Figure 3c). Interestingly, normalized parallel mutation counts from resistant cultivar under elevated O_3_ were comparable to that from susceptible cultivar under ambient and elevated O_3_. We classified de novo parallel mutations into (i) those retained from mid to end‐season, indicating their selection throughout the growing season, (ii) those that appeared after mid‐season, and (iii) those that were possibly deleterious since they appeared in the mid‐season but were not retained at the end‐season (Figure 3c). Owing to the low sequencing depth, some de novo parallel mutations observed in population from resistant cultivar could not be assigned to either of these categories. We observed a lower proportion (normalized counts, i.e. N, refer to Figure 3c, (i)) of de novo parallel mutations retained from mid‐to‐end season and a greater proportion of deleterious mutations (refer to N in Figure 3c, (iii)) under elevated O_3_ irrespective of the host cultivar. These findings suggest that the presence of elevated O_3_ may favor random mutations in the pathogen population.

### 3.4 Signatures of Parallel Evolution in the Pathogen Population

The above observations led us to annotate the signatures of parallel evolution, that is, SNVs observed independently across different treatments. Thus, the sites retained over time (from the mid‐season to the end‐season, belonging to Figure 3c category (i) possibly selected sites) in the pathogen population were then compared for their presence across host genotypes and O_3_ conditions, both in terms of their site ids (i.e., at the same position) and the associated same gene id spanning the mutation sites (Figure S6a). We identified 25 sites that spanned 12 genes that showed the presence of parallel de novo mutation in all the treatments regardless of host genotype and O_3_ conditions (Figure S6b). These sites were predominantly associated with signaling pathways known to play a crucial role in virulence, aiding pathogens in promoting disease within the host (Table S5; Li et al. 2019; Wang and Qian 2019). There were 72 sites spanning 35 genes carrying de novo mutation in the pathogen population from susceptible cultivar common in both environments (Figure S6b). These included genes related to overall pathogen fitness, including those related to DNA repair photolyase, the outer membrane protein beta‐barrel family, the multidrug efflux RND transporter permease subunit, and peptidoglycan biosynthesis (Table S5). Six sites spanning one gene and two intergenic regions were uniquely identified as selected sites in the population recovered from the resistant cultivar under the ambient environment. These genes encode the chemotaxis protein and the monovalent cation proton antiporter 2 transporter (TC 2.A.37) family. Three de novo mutation sites retained from the mid‐season to the end‐season on resistant cultivar under elevated conditions were also found on susceptible cultivar in both environments. These sites are located in the intergenic region of two genes, one encoding cache2/3 fusion domain‐containing protein, which may help the pathogen locate nutrients under epiphytic conditions on plant leaves, identify potential entry points into host cells (Brewster et al. 2016) and the second encoding dienelactone hydrolase involved in chlorocatechol degradation (Schlömann et al. 1993). These findings are of interest given the observation of greater disease severity on resistant cultivar under elevated O_3_. There were parallel de novo mutations under elevated O_3_, linked to genes such as the sulfotransferase family, peroxiredoxin activity, and carboxymuconolactone decarboxylase family protein, known for an antioxidative response in the host (Table S5) (Chen et al. 2015; Kamariah et al. 2018; Mougous et al. 2002). We found one unique mutation in the pathogen population from a resistant cultivar under elevated O_3_ only by the end‐season (belonging to Figure 3c category (ii)), which was related to an intergenic region of genes known as the TldD protein, identified under different stresses (Vobruba et al. 2022; Table S6).

### 3.5 Strong Selective Pressure on Selected Loci

The parallel mutations identified in Section 3.3 (evolutionary modifications, Figure 3) were further analyzed to determine if the associated DNA sequences evolved neutrally. We used the PoPoolation tool to compute Tajima's *D* with a 1‐kb window size. Regions containing these mutations (from Section 3.3) were then selected. Due to low coverage data for the majority of the sites in case of resistant cultivar during mid‐season, only end‐season data were considered. Overall, a higher number of regions exhibited positive Tajima's *D* values in populations from the resistant cultivar under ambient conditions compared to susceptible cultivars under both environmental conditions and resistant cultivars under elevated conditions (Figures S7 and S8). However, more regions had negative Tajima's *D* values in populations from the resistant cultivar under elevated O_3_ conditions. Interestingly, specific regions exhibited extreme Tajima's *D* values greater than 2 or less than −2 under different conditions, suggesting strong selection pressure in these areas. Highly positive values indicate balancing selection, characterized by a reduced occurrence of rare alleles, while highly negative values point to purifying selection, marked by an increased presence of rare alleles. One region (Tajima's *D* = −2.006) was identified in the population from a resistant cultivar under ambient conditions, associated with the AMP‐binding protein gene. In contrast, in populations from susceptible cultivars, one genomic region displayed extremely positive Tajima's *D* values (*D* = 2.53 and 3.03 under ambient conditions and elevated O₃, respectively) linked to the trypsin‐like serine protease‐coding gene. Moreover, 29 windows with extremely negative Tajima's *D* values (*D* < −2) were observed in populations from susceptible cultivars under normal conditions, associated with genes encoding type VI secretion system core protein TssA, XopAD, TonB‐dependent receptors, and other proteins. Similarly, under elevated O₃ conditions in susceptible cultivars, 10 windows exhibited extreme negative values linked to genes encoding phosphoenolpyruvate synthase, conjugative proteins, lytic murein transglycosylase B, and transporters (Table S7A).

Notably, some regions demonstrated opposing trends, showing positive values under certain conditions and negative values under others. Specifically, four regions displayed negative Tajima's *D* values under the combined stress of resistance and elevated O_3_ stress, while other treatments indicated neutral evolution. These regions are associated with genes coding for *YceI family protein*, *cytochrome b*, *sulfotransferase*, and *histidine‐type phosphatase*. Additionally, five windows (spanning nine genes) exhibited negative Tajima's *D* values only under elevated O_3_ conditions, regardless of host type. These genes were linked to transport and signaling proteins, enzymes, catalytic proteins and hypothetical proteins. Similarly, 7 regions with positive values (spanning 12 genes) and 7 regions with negative values (spanning 12 genes) were observed in populations from resistant hosts irrespective of conditions, where the susceptible host exhibited positive and neutral selective pressures in those regions, respectively. These regions could be significant for the resistant host, as they include genes coding for *type VI secretion core proteins*, *TonB‐dependent receptors*, and *type III effector proteins*. Furthermore, five regions (spanning eight genes) showed positive values exclusively in the resistant host under ambient conditions, while the other treatments displayed negative values in these regions. These regions included genes coding for type VI core proteins, enzymes, response regulator transcription factor, and ATPase factor (Table S7B).

### 3.6 A Relatively High Rate of Gene Flux Events Was Observed in the Pathogen Population During Adaptation to Resistant Cultivar

**FIGURE 4:**
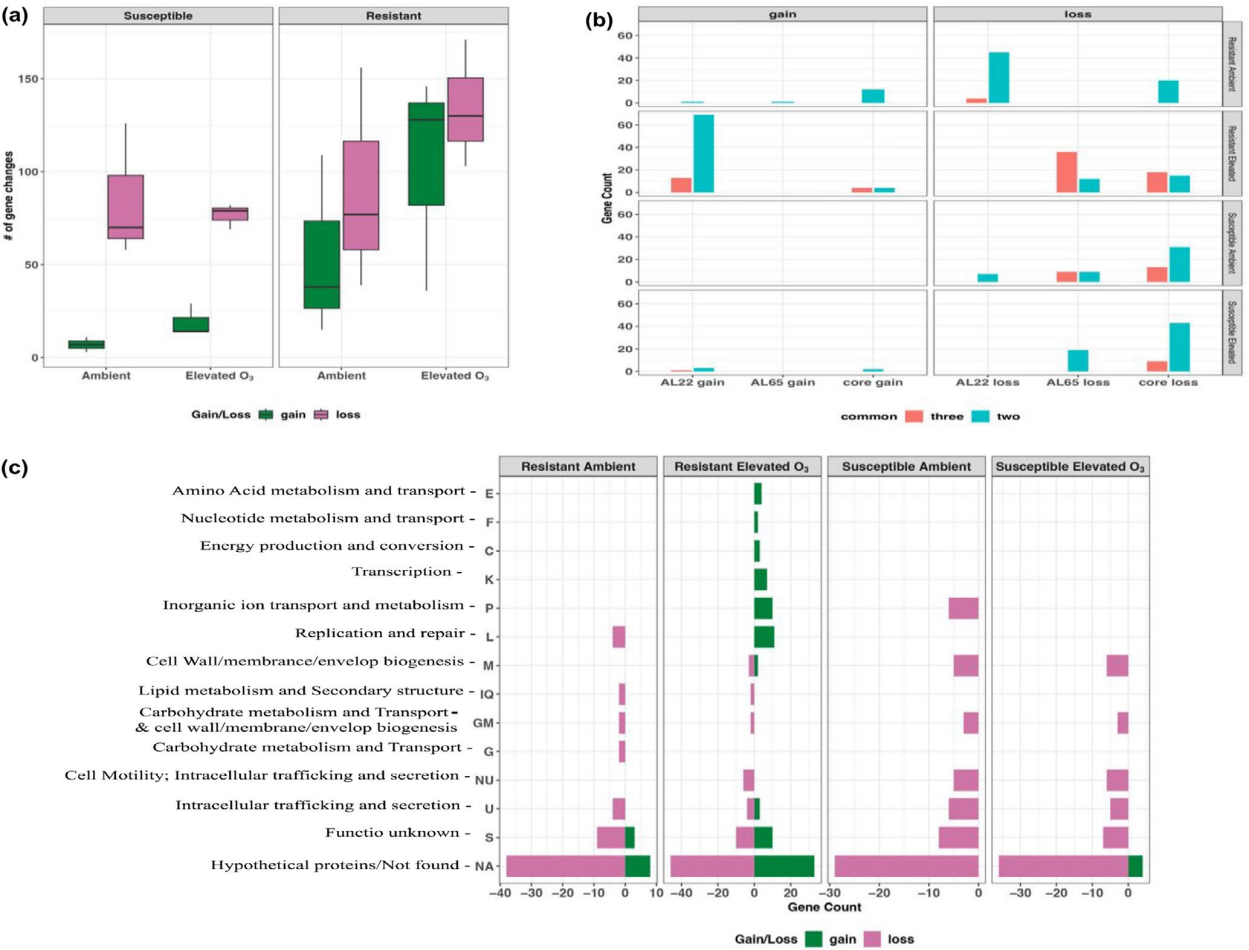
Gene flux in the pathogen population. (a) Boxplot showing the number of gene gains (absent during the mid‐season and present by the end‐season in the pathogen population) and gene losses (present during mid‐season and lost by the end‐season) across all the chambers. (b) Bar plot presenting the number of gene changes common among two and three chambers/replicates of different treatments. The core genes here refer to those common to both strains, and genes unique to each strain are indicated as AL65 gain/loss or AL22 gain/loss. (c) Bar plot showing the Cluster of Orthologous Groups (COG)‐based functional profiling of gene changes common in two and three chambers across different treatments.

Next, we measured changes in the gene pool, indicating variations in pangenome size. Our hypothesis posited that selection pressure would induce alterations in the pathogen gene pool, favoring gene acquisition or loss for improved pathogen fitness; thus, we anticipated more gene gain/loss events in the pathogen populations under both stressors. We defined “gene gain” as a gene reappearing from mid‐to‐end‐season and “gene loss” as a gene present in mid‐season but lost by the end‐season. A threshold of copy number of 0.35 was considered for determining gene presence (Garud and Pollard 2020). We observed a higher gene flux in the pathogen population from resistant cultivars, especially under elevated O_3_ conditions. However, there were no differences in gain/loss events in the pathogen population from susceptible cultivars irrespective of environmental conditions (Figure 4a). We identified a total of 90 gene gain events and 81 gene loss events in the resistant cultivar under elevated O_3_, out of which gene gain events occurred primarily in AL22, and losses occurred in the AL65 genome (Figure 4b). We found evidence of parallel gene flux events, that is, those observed across all three replicates. Approximately 20 core genes were lost in the pathogen population from the resistant cultivar under elevated O_3_. Around 8–10 core genes were lost in pathogen population from the susceptible cultivar from either environment. Interestingly, genes belonging to the COG categories M, GM, N, U, and P, which are related to metabolism, transport, cell wall, cell motility, and intracellular trafficking, were lost from the susceptible cultivar. In contrast, M and U were gained in resistant cultivar under elevated O_3_. Additionally, genes belonging to the C, P, K, and L categories were gained in the pathogen population from resistant cultivars under elevated O_3_ conditions, with adaptive advantages in terms of energy production, transcription, nucleotide metabolism, replication, and repair (Figure 4c). Although seen as a lack of parallel genes being lost from pathogen population from resistant cultivar under ambient conditions, this may have been an artifact due to the low sequencing depth for some resistant cultivar samples (Figure 4b).

## 4 Discussion

Global climate change can affect infectious diseases in a nonlinear fashion. However, we have little evidence that climate change has already led to a surge of infectious diseases as the globe has warmed up over the past century. Models have contrasting predictions of increases in the geographic range of infectious diseases, range shifts in disease distributions (Lafferty 2009), or overall increased pathogen virulence (Velásquez, Castroverde, and He 2018), thus, demanding empirical studies conducted to evaluate the impact of climatic shifts on disease dynamics under future climatic conditions. Rapid and gradual changes in the environment can affect multiple scales of plant‐pathogen interactions from the molecular level to the community level, with feedback linking across these scales and adding uncertainty to the outcomes of resistance management. In this study, we observed higher disease severity and impaired physiological response on resistant cultivars of pepper under elevated O_3_ conditions in an OTC field setup. Given this observation, our goal was to understand gene to population level response of the pathogen when challenged with host defense and abiotic stress simultaneously. We harnessed the power of metagenomics to monitor the generation and maintenance of genetic variation in the pathogen population in response to altered climate (elevated O_3_) and host selection pressure, its impact on phenotypic changes on which natural selection acts. The experimental setup involving OTCs allowed us to gather empirical evidence on rapid evolution in a single growing season under future climatic conditions.

We observed that elevated O_3_ levels did not influence disease severity on susceptible cultivar and did not alter pathogen population structure. A single pathogen genotype, AL65, dominated the susceptible cultivar throughout the growing season. While the resistant cultivar supported the coexistence of both pathogen genotypes throughout an ambient environment, the strain turnover was observed under elevated O_3_, with AL65 being dominant during mid‐season, followed by the coexistence of both strains by the end‐season. This observation is similar to the seasonal turnover of specific pathogen genotypes with dominance depending on their fitness levels, as observed for pseudomonads isolated from sugar beet leaves over the course of three growing seasons (Ellis, Thompson, and Bailey 1999). The colonization success of AL65 on the resistant cultivar during mid‐season was linked to higher disease severity under elevated O_3_, although accompanied by high variation (Figure 1). The altered pathogen dynamics on the resistant cultivar under elevated O_3_ could be a result of either altered host selection pressure or altered ecological interactions among the two closely related strains or altered interaction of individual strains with the host with changing host defense response under abiotic stress (Castelijn et al. 2012; Papkou et al. 2021; Yang, Ma, and Zu 2022). Interestingly, despite the end‐season pathogen population being heterogeneous in both environments, phenotypic differences were evident in the two environments, with resistant plants under elevated O_3_ showing a 2% increase in disease severity compared to ambient conditions. Since the resistant cultivar used in this study contained *bs5* resistance recessive gene; thus, a small increase in disease severity ratings in a single growing season may be indicative of slow erosion. While the absolute abundance of *Xanthomonas* could not solely explain this difference (Bhandari et al. 2023), we investigated whether the amount of genetic variation available for selection to act on differed across environments and whether new alleles of large effect could be linked to the phenotype. The presence of a mixed pathogen population and the maintenance of heterogeneity in response to stressors has been referred to as a mechanism of rapid evolution resulting in increased pathogen fitness (Aertsen and Michiels 2005; Balaban et al. 2004; Caballero‐Huertas et al. 2023; Dhar and McKinney 2007; Hiramatsu et al. 2001; Lidstrom and Konopka 2010; Longo and Hasty 2006; Schröter and Dersch 2019). This coexistence may suggest that the interplay of two pathogen genotypes may contribute to the adaptation to small‐effect QTL on the resistant cultivar.

We found that elevated O_3_ alone was not the primary driver of genetic differentiation in the pathogen population, as suggested by the stable within‐host nucleotide diversity of *Xp* on susceptible cultivars under both environments. However, host selection pressure on the resistant cultivar led to relatively greater nucleotide diversity. The elevated O_3_ did not affect the average level of polymorphism. Elevated O_3_ also supported high variation in the levels of polymorphism on the resistant cultivar, signifying the diversity of genetic variation available for adaptation over time (Figure 2). This higher polymorphism may suggest an adaptive strategy in the presence of stressors, as observed in various biological systems such as the intestinal microbiota, coral reefs, marine life, and wildlife populations (Candela et al. 2012; Bonachela, Choua, and Heath 2022; Leray et al. 2021; Reusch 2014; Risely et al. 2023; Torda et al. 2017). However, higher polymorphism may not always be correlated with beneficial adaptive mutations, as stress‐induced maladaptive changes have also been reported in other systems (Aggeli, Li, and Sherlock 2021; Galhardo, Hastings, and Rosenberg 2007; Metzgar and Wills 2000; Sprouffske et al. 2018).

Strong purifying selection in the pathogen population was observed irrespective of cultivar and conditions, acting against newly emerged harmful mutations, preserving the genetic traits, and leaving imprints on genetic diversity by altering the distribution of genetic variants at specific sites (Figure 3; Cvijović, Good, and Desai 2018). The presence of elevated O_3_ led to evidence of positive selection in the pathogen population on susceptible cultivar in genes encoding transcriptional regulators, sigma factors, and TrmH (methyltransferase involved in posttranslational regulation influencing the oxidative stress response, and suppression of host immune response (Figure 3; Galvanin et al. 2020; Rimbach et al. 2015)). The evidence of selection was not limited to the nucleotide level, but selection at the gene level with parallel gene gain/loss events was evident. Variations in the rates of gene gain and loss affect pathogen's fitness (Brockhurst et al. 2019; Domingo‐Sananes and McInerney 2021; Lefébure and Stanhope 2007; Moulana et al. 2020). Pathogen adopted a “less is more” strategy when adapting to resistant cultivar and under elevated O_3_ conditions that led to more gene loss events (Figure 4; Li et al. 2017; Seidl and Thomma 2017; Simonsen 2022). This observation of the coexistence of both pathogen genotypes and more gene loss events on resistant cultivar also aligns with the black queen hypothesis, which states that species turnover and interactions among members in a diverse community select for a loss of genes redundant in function and promote interdependence (Morris, Lenski, and Zinser 2012).

While we observed evidence of a high level of polymorphism and signatures of positive selection in response to elevated O_3_ or host selection pressure, we categorized the observed genetic variations into those arising from standing genetic variation (those observed due to ecological changes resulting in strain‐specific allele shifts) and those from evolutionary modifications via retention of parallel de novo mutations throughout the growing season. Here, we focused on short‐term single‐season within‐host changes in pathogen populations, which may provide insights into pathogen adaptation over longer time scales. The fact that we observe evidence of parallel evolution with allele shifts or de novo mutations that are retained over the growing season across replicates suggests that short‐term evolutionary forces could contribute to the signals of adaptation that accumulate over the long term. For example, parallel evolutionary signatures in genes specifically implicated in functions to overcome host defense, ROS, or enhanced nutrient assimilation might contribute to the continuum responsible for resistance erosion in the longer term. We find that both standing genetic variation and de novo mutations play a role in the rapid adaptation of pathogen onto quantitative resistant cultivar, although the contribution of each of these mechanisms of variation was variable depending on O_3_ environment. As strain turnover was observed under elevated O_3_ conditions, a larger contribution of standing genetic variation was evident (Figure 3). Examples of standing genetic variation in response to stressors contributing to rapid evolution through small allele frequency changes at multiple loci are common across prokaryotes or eukaryotes (Chen and Garud 2022; Dayan et al. 2019). We observed signatures of parallel de novo mutations in the pathogen population recovered from resistant cultivar under ambient condition. Many of these parallel de novo mutations were retained throughout the growing season. Resistant hosts are hypothesized to impose stronger selection pressure leading to host–pathogen arms race, and many of such studies have been biased toward pathogen virulence/effector genes (Alfano and Collmer 1996; Arnold et al. 2007; Gassmann et al. 2000; Ma et al. 2006; Pitman et al. 2005; Trivedi and Wang 2014). In our study, we observed parallel evolution with mutations independently evolving in pathogen populations recovered from different chambers/resistant plants, some of which were independently targeting the same gene. These results indicate that these mutations did not evolve by random drift and can be targets of strong selection pressure, as evidenced by Tajima's *D* values, refuting the null hypothesis of neutral evolution (Table S7, Figures S7 and S8; Tajima 1989). Although observed parallel across treatments, and with signature of selection, and retained over the course of growing season, these mutations cannot be assumed to be adaptive, and further experiments with recovered pathogen isolates from the resistant cultivar would be needed. Such experiments may include inoculating them individually on resistant and susceptible cultivars and evaluating whether the isolates with these specific mutations show increased disease severity on resistant cultivar. Such empirical validation will be useful to categorize these mutations as beneficial or maladaptive mutations. Alternatively, a reverse‐genetics approach by introducing targeted mutations in wild‐type strains to test for the fitness effects of these retained mutations on resistant cultivar may help understand the adaptation process to quantitative resistance. Interestingly, although we observed a greater proportion of SNVs on resistant cultivar under elevated O_3_, many of those SNVs were observed due to allele shift, as explained by strain turnover. Some de novo mutations did not persist throughout the growing season, suggesting possible deleterious mutations, or lacked signatures of parallel evolution across replicates under elevated O_3_ conditions. These greater proportions of transient mutations may explain high degree of variation observed in disease severity levels across replicates under elevated O_3_. These findings suggest that abiotic stressors may have an influence in altering pathogen evolution with unpredictable outcomes. The observation of overall lower proportion of de novo mutations in response to elevated O_3_ on resistant cultivar may be the result of altered host selection pressure compared to that under ambient environment. Indeed, the host transcriptome data indicate compromised immune response of resistant cultivar under elevated O_3_ (Modelski, unpublished). Multi‐seasonal experiments under OTC conditions by subjecting pathogen to altered climatic conditions are needed to understand the fate of these transient mutations in terms of their fixation and whether these small effect loci contribute toward further erosion of quantitative resistance. In summary, the patterns of genetic variation observed in this study may help in predicting the evolution of the pathogen and predicting the durability of disease resistance. However, the finding of random transient mutations leading to increased within‐host polymorphism may point to difficulties in predicting pathogen evolution under climatic shifts.

## 5 Future Directions

As we navigate the complex interdependent plant–pathogen–environment interactions, climate sensitivity of plant resistance genes and changing pathogen population structure are important parameters to consider for plant breeding programs. Field‐experimental setup, such as OTCs used in this study can be useful to understand the responses of disease outbreaks at finer scale of nucleotide level (from population to SNP level) under future climatic conditions, while also accounting for inter‐seasonal variability. Experiments involving multiple host generations across multiple seasons would be ideal to understand how host–pathogen evolutionary arms race will be shaped by shifts in the climatic patterns. While our understanding to date on pathogen population structure is guided by large‐scale isolate genome sequencing efforts, this approach can overlook low abundant pathogen variants in the population, some of which may have higher fitness under changing environmental conditions. Thus, using methods such as strain‐resolved metagenomics and applying population genomics methods on spatiotemporal data, as applied in this study, can allow us to study evolution in action. This present work indicated that pathogen population responds to the altered climate and host defense by maintaining heterogeneity in the population, but also responding through evolutionary modifications, including new mutations, although many of these mutations were not parallel and not retained under altered climatic conditions, hinting toward plasticity, but also unpredictability of the fate of the pathogen. Short‐term studies like this one when combined with phenotyping of pathogen variants recovered from the resistant plants under elevated ozone can have potential to inform on the virulence potential of the adapted pathogen and perhaps, in providing much needed data that will form a basis for theoretical models predicting host–pathogen interactions under climate change.

## Conflicts of Interest

The authors declare no conflicts of interest.

## Supporting information


**Figure S1.** Ozone (O_3_) levels throughout the season within the open‐top chambers were on average 29.33 ppb (parts per billions) for Ambient chambers and 87.65 ppb for elevated O_3_ chambers. O_3_ levels above 40 ppb are considered to be highly phytotoxic (Saxena et al., 2020).
**Figure S2.** Points in the figure show the Absolute abundance of *Xp* genotypes compared to disease severity during the mid and end‐season sampling time points for the susceptible cultivar under ambient versus elevated O_3_ levels. The vertical lines present the averages of the mid and end‐season disease severity for ambient and elevated O_3_ levels.
**Figure S3.** Absolute abundance (ng DNA per mg of leaf tissue) of *Xp* genotypes as averages in the leftmost circular chord diagram and as raw values in the rightmost barplots.
**Figure S4.** Plot showing average nucleotide diversity (*π*) of *Xp* population for each chamber.
**Figure S5.** Barplot indicating within host polymorphism with SNV counts (single nucleotide variant) for each chamber. These counts consist of different types of mutations (1D, 2D, 3D, and 4D) having an allele frequency between 0.2 and 0.8, where a site with 1D is one in which an amino acid change caused by nucleotide difference (non‐synonymous), while a site with a 4D cannot be caused by any nucleotide difference (synonymous), and 2D & 3D indicates the either two or three possible changes, respectively can be tolerated, before an amino acid is altered (Chen and Garud, 2022; Nayfach, 2015/2022).
**Figure S6.** (a) Visual graphics for comparisons when the same site hit in parallel across different treatments; when the same gene was mutated across different treatments irrespective of SNV location; (b) Table at the bottom includes counts related to the SNVs that retained from mid‐season to the end‐season in population. These counts are based on when parallel de novo mutation appeared across different treatments based on the same SNV site and when the same gene belonging to parallel *de novo* mutations was mutated across different treatments.
**Figure S7.** Tajima’s *D* calculated for those regions where different sites (from Section 3.3) were located in the genome. Each dot is presenting those 1000 bp regions around each site/SNP location in the genome of pathogen.
**Figure S8.** Counts of different 1000 bp regions from Figure S7, signifying if different treatment is under different selection pressures. These are only for end‐season.

**Table S1.** Microbial species identified as resident microbial community members, with mean abundance of more than 0 within the samples.
**Table S2.** List of blacklisted genes from Xp pangenome‐ these are identified as having high homology (97% or above identity based on BLAST analysis against genes from phyllosphere community members).
**Table S3.** Reads counts before and after removing blacklisted genes from samples.
**Table S4.** Parallel sites (across replicates) belonging to Allele‐shift or Standard genetic variations (with < 0.2 allele frequency during mid‐season and became > 0.8 by the end‐season).
**Table S5.** Parallel *de novo* mutation sites appeared across different conditions at the same position in the pathogen population genome. These are possibly selected de novo mutations (belonging to category (i)).
**Table S6.** Parallel *de novo* mutation sites across different conditions based on the same SNV site in the pathogen genome. These include all the de novo mutations present at the end season (both category (i), (ii), & (iv)).
**Table S7.** (A) Region from the selected loci showing extreme Tajima’s *D* values. (B) Tajima’s *D* shown for different treatments showing opposite trends during end season.

## Data Availability

Sequence data generated from this work have been deposited in the SRA (Sequencing Read Achieve) database under the BioProject accession PRJNA889178. *Xp* strain AL22 has been submitted under BioProject accession PRJNA1077988. *Xp* AL65 used from submission under BioProject accession PRJNA953417. All the scripts, input files, and output files used in this study are available on Zenodo repository (https://doi.org/10.5281/zenodo.14623261).
