# Supplementary material for: Navigating Host Immunity and Concurrent Ozone Stress: Strain‐Resolved Metagenomics Reveals Maintenance of Intraspecific Diversity and Genetic Variation in Xanthomonas on Pepper
Source: Evol Appl. 2025 Jan 14;18(1):e70069. doi: 10.1111/eva.70069 (PMC11732741; doi:10.1111/eva.70069)
Supplement: Supplementary file 1 — Figure S1. Ozone (O3) levels throughout the season within the open‐top chambers were on average 29.33 ppb (parts per billions) for Ambient chambers and 87.65 ppb for elevated O3 chambers. O3 levels above 40 ppb are considered to be highly phytotoxic (Saxena et al., 2020). Figure S2. Points in the figure show the Absolute abundance of Xp genotypes compared to disease severity during the mid and end‐season sampling time points for the susceptible cultivar under ambient versus elevated O3 levels. The vertical lines present the averages of the mid and end‐season disease severity for ambient and elevated O3 levels. Figure S3. Absolute abundance (ng DNA per mg of leaf tissue) of Xp genotypes as averages in the leftmost circular chord diagram and as raw values in the rightmost barplots. Figure S4. Plot showing average nucleotide diversity (π) of Xp population for each chamber. Figure S5. Barplot indicating within host polymorphism with SNV counts (single nucleotide variant) for each chamber. These counts consist of different types of mutations (1D, 2D, 3D, and 4D) having an allele frequency between 0.2 and 0.8, where a site with 1D is one in which an amino acid change caused by nucleotide difference (non‐synonymous), while a site with a 4D cannot be caused by any nucleotide difference (synonymous), and 2D & 3D indicates the either two or three possible changes, respectively can be tolerated, before an amino acid is altered (Chen and Garud, 2022; Nayfach, 2015/2022). Figure S6. (a) Visual graphics for comparisons when the same site hit in parallel across different treatments; when the same gene was mutated across different treatments irrespective of SNV location; (b) Table at the bottom includes counts related to the SNVs that retained from mid‐season to the end‐season in population. These counts are based on when parallel de novo mutation appeared across different treatments based on the same SNV site and when the same gene belonging to parallel de novo mutations was mutat [file EVA-18-e70069-s002.docx]

**Supplementary file 1**

**Navigating host immunity and concurrent ozone stress: Strain-resolved metagenomics reveals maintenance of intraspecific diversity and genetic variation in *Xanthomonas* on pepper**

Running title: Pepper-*Xanthomonas* arms race under climate change

**Figure S1.** Ozone (O_3_) levels throughout the season within the open-top chambers were on average 29.33 ppb (parts per billions) for Ambient chambers and 87.65 ppb for elevated O_3_ chambers. O_3_ levels above 40 ppb are considered to be highly phytotoxic [111].

**Figure S2.** Points in the figure show the Absolute abundance of *Xp* genotypes compared to disease severity during the mid and end-season sampling time points for the susceptible cultivar under ambient versus elevated O_3_ levels. The vertical lines present the averages of the mid and end-season disease severity for ambient and elevated O_3_ levels.


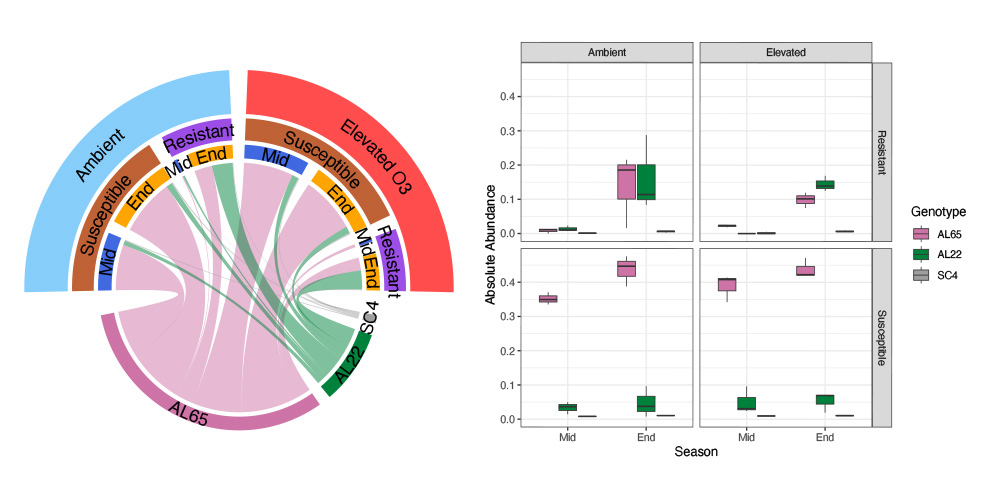


**Figure S3.** Absolute abundance (ng DNA per mg of leaf tissue) of *Xp* genotypes as averages in the leftmost circular chord diagram and as raw values in the rightmost barplots.

**Figure S4.** Plot showing average nucleotide diversity (π) of *Xp* population for each chamber.

**Figure S5.** Barplot indicating within host polymorphism with SNV counts (single nucleotide variant) for each chamber. These counts consist of different types of mutations (1D, 2D, 3D, and 4D) having an allele frequency between 0.2 and 0.8, where a site with 1D is one in which an amino acid change caused by nucleotide difference (non-synonymous), while a site with a 4D cannot be caused by any nucleotide difference (synonymous), and 2D & 3D indicates the either two or three possible changes, respectively can be tolerated, before an amino acid is altered (D. W. Chen & Garud, 2022; Nayfach, 2015/2022).

**Figure S6. (a)** Visual graphics for comparisons when the same site hit in parallel across different treatments; when the same gene was mutated across different treatments irrespective of SNV location; **(b)** Table at the bottom includes counts related to the SNVs that retained from mid-season to the end-season in population. These counts are based on when parallel de novo mutation appeared across different treatments based on the same SNV site and when the same gene belonging to parallel de novo mutations was mutated across different treatments.

**Figure S7.** Tajima’s D calculated for those regions where different sites (from section 3.3) were located in the genome. Each dot is presenting those 1000bp regions around each site/SNP location in the genome of pathogen.

**Figure S8.** Counts of different 1000bp regions from Figure S7, signifying if different treatment is under different selection pressures. These are only for end-season.
